# Estimation of Joint Moments During Turning Maneuvers in Alpine Skiing Using a Three Dimensional Musculoskeletal Skier Model and a Forward Dynamics Optimization Framework

**DOI:** 10.3389/fbioe.2022.894568

**Published:** 2022-06-24

**Authors:** Dieter Heinrich, Antonie J. Van den Bogert, Werner Nachbauer

**Affiliations:** ^1^ Department of Sport Science, University of Innsbruck, Innsbruck, Austria; ^2^ Department of Mechanical Engineering, Cleveland State University, Cleveland, OH, United States

**Keywords:** skiing, turning maneuver, joint moments, forward dynamics, optimal, control, data tracking, musculoskeletal model

## Abstract

In alpine skiing, estimation of the joint moments acting onto the skier is essential to quantify the loading of the skier during turning maneuvers. In the present study, a novel forward dynamics optimization framework is presented to estimate the joint moments acting onto the skier incorporating a three dimensional musculoskeletal model (53 kinematic degrees of freedom, 94 muscles). Kinematic data of a professional skier performing a turning maneuver were captured and used as input data to the optimization framework. In the optimization framework, the musculoskeletal model of the skier was applied to track the experimental data of a skier and to estimate the underlying joint moments of the skier at the hip, knee and ankle joints of the outside and inside leg as well as the lumbar joint. During the turning maneuver the speed of the skier was about 14 m/s with a minimum turn radius of about 16 m. The highest joint moments were observed at the lumbar joint with a maximum of 1.88 Nm/kg for lumbar extension. At the outside leg, the highest joint moments corresponded to the hip extension moment with 1.27 Nm/kg, the knee extension moment with 1.02 Nm/kg and the ankle plantarflexion moment with 0.85 Nm/kg. Compared to the classical inverse dynamics analysis, the present framework has four major advantages. First, using a forward dynamic optimization framework the underlying kinematics of the skier as well as the corresponding ground reaction forces are dynamically consistent. Second, the present framework can cope with incomplete data (i.e., without ground reaction force data). Third, the computation of the joint moments is less sensitive to errors in the measurement data. Fourth, the computed joint moments are constrained to stay within the physiological limits defined by the musculoskeletal model.

## 1 Introduction

In alpine skiing, field experiments in the natural environment (i.e., on the ski slope) are essential to analyze the movement of the skier regarding performance characteristics or for the purpose of injury prevention. While performance analyses primarily focus on kinematic characteristics of the skier such as the trajectory of the center of mass, the skier’s velocity and/or the path length of a turning maneuver (e.g., Federolf, 2012; Spörri et al., 2012; Gilgien et al., 2015; Fasel et al., 2018a), kinetic characteristics such as the joint moments at the lumbar, hip, knee and ankle joints of the skier or the ground reaction forces are the main focus in the context of injury prevention (e.g., Stricker et al., 2010; Klous et al., 2012; Lee et al., 2017; Spörri et al., 2018; Meyer et al., 2019).

Focusing on injury prevention, inverse dynamics is the preferred approach to estimate the joint moments acting on a skier during turning maneuvers (e.g., van den Bogert et al., 1999; Klous et al., 2012, 2014; Hirose et al., 2013; Lee et al., 2017). An inverse dynamics analysis is typically based on kinematic data of the body segments of the skier as well as measurement data of external forces (i.e., ground reaction forces) as input and provides the net joint moments of the skier as output (Winter 2009). The loading of the knee joint is of high interest, since most serious injuries in recreational skiing (Posch et al., 2021) and competitive alpine skiing are located at the knee (Haaland et al., 2016; Barth et al., 2021). Inverse dynamics is computationally inexpensive, straightforward and available in several software packages such as OpenSim or Anybody. However, it has some important limitations. First, in an inverse dynamics analysis the kinematics and ground reaction forces are dynamically not consistent (Fluit et al., 2014). This inconsistency arises due to measurement errors of the kinematics and ground reaction forces as well as differences between the biomechanical model used in the inverse dynamics analysis and the real physical system (Hatze, 2002) and introduces errors in the computation of the joint moments (Faber et al., 2018). Second, an inverse dynamics analysis requires double differentiation of the segment kinematics, which amplifies errors in the measurement data. Consequently, inverse dynamics is highly sensitive to measurement errors (Cahouët et al., 2002; Pàmies-Vilà et al., 2012). Third, the computed joint torques are not constrained to stay within physiological limits (Bailly et al., 2021). The main reason is that muscle characteristics such as the maximum isometric force, the force-length relationship, the force-velocity relationship and the activation dynamics are not taken into account in the inverse dynamics analysis of the joint moments. Consequently, the estimated joint moments might be unrealistic high and physiologically not plausible (Bailly et al., 2021).

These limitations of inverse dynamics analysis may have affected previous studies in alpine skiing estimating joint moments during turning maneuvers. Hirose et al. (2013), for example, computed the joint moments at the lower extremities during a carving turn using an inverse dynamics analysis that was based on kinematic data obtained by an IMU based system and measured ground reaction forces between the ski boot and ski. They reported a peak external hip flexion moment of about 900 Nm, which is about a factor of 2.5 above the maximum voluntary hip joint torque reported in the study of Anderson et al. (2007) for the age group 19 to 25. Furthermore, Klous et al. (2012), computed knee extension moments up to 8.35 Nm/kg and 4.07 Nm/kg for a skidded and a carved turn, respectively. Although the speed of the skier was relatively low in the carved turn (v = 13.9 m/s) and the skidded turn (v = 10.4 m/s), the reported peak knee flexion moments exceeded in turn the maximum voluntary joint torques derived by Anderson et al. (2007). The inverse dynamics analysis incorporated kinematic data captured by a multi-camera system and ground reaction force measured by custom-built mobile force platforms mounted between the ski binding and the ski. In addition, Klous et al. (2012) reported peak external knee abduction moments, which are about a factor of three higher than the assumed injury threshold of 125 Nm valgus moment in the study of McLean et al. (2008), although they did not investigate an injury prone situation. Thus, the reported peak joint moments in these studies are likely to be error prone and unrealistic high.

As an alternative to inverse dynamics, recent advances in forward dynamics methods opened up new opportunities (Erdemir et al., 2007). Specifically, given a musculoskeletal model, forward dynamics optimization such as forward dynamics assisted data tracking offer the possibility to estimate dynamically consistent kinematics and ground reaction forces as well as joint torques and muscle forces (Nitschke et al., 2020). In addition, these methods are less sensitive to errors in the measurement data and allow to incorporate the force generating muscle properties such as the maximum isometric force, the force-length relationship, the force-velocity relationship and the muscle activation dynamics (Erdemir et al., 2007).

In alpine skiing, only a few studies applied a musculoskeletal simulation model in combination with forward dynamics optimization to estimate consistent joint kinematics and ground reaction forces, joint torques and muscle forces (e.g., Gerritsen et al., 1996; Heinrich et al., 2014, 2018). However, all of these studies incorporated a two dimensional model of an alpine skier, which was constrained to the sagittal plane and applied to analyze jump landing maneuvers in downhill skiing. Analyzing turning maneuvers, however, requires a three dimensional skier model. To the authors’ knowledge, no three dimensional musculoskeletal skier model has been developed. Therefore, the first objective of the present study was to develop a three dimensional musculoskeletal model of an alpine skier capable of simulating turning maneuvers. The second objective was to apply the musculoskeletal skier model in combination with a forward dynamics optimization framework to estimate dynamically consistent kinematics, ground reaction forces and joint moments during a turning maneuver. The estimation of the joint moments was constrained such that computed joint torques stayed within physiological limits imposed by the musculoskeletal model.

## 2 Materials and Methods

### 2.1 Musculoskeletal Skier Model

**FIGURE 1:**
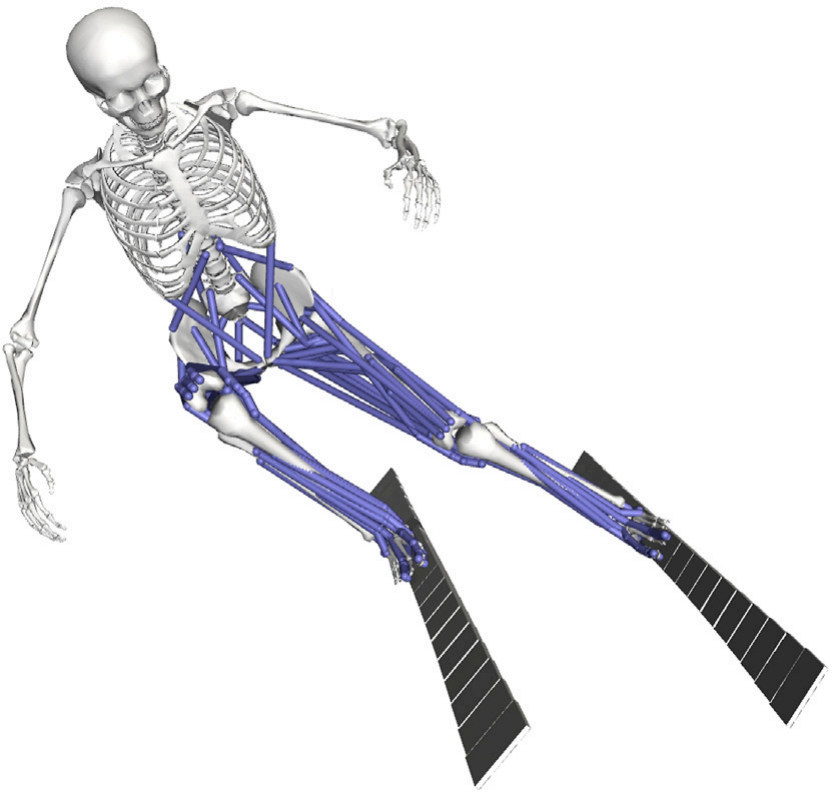
Three dimensional musculoskeletal model of the skier with two skis, 53 kinematic degrees of freedom (19 for the skier and 17 for each ski) and 94 muscles visualized in OpenSim (Delp et al., 2007). The subtalar and mtp joints were locked because of the skier’s ski boots, which allowed only plantarflexion and dorsiflexion at the ankle joints.

We developed a three dimensional musculoskeletal model of an alpine skier with two skis and 53 degrees of freedom (19 for the skier and 17 for each ski) to simulate turning maneuvers in alpine skiing (Figure 1). The skeletal model of the skier consisted of 20 rigid segments and was derived from the full-body OpenSim model of Hamner et al. (2010). At each lower extremity the subtalar and mtp joints were locked because of the skier’s ski boot, which allows only plantarflexion and dorsiflexion at the ankle joint. The restraining effect of the ski boot was represented by a passive moment at the ankle joint incorporating the non-linear relation between the boot-induced moment and the ankle joint angle (Eberle et al., 2017). To increase computational speed the position of the arms of the skier was locked in a typical position and the mass of the ski poles was neglected. In total, the skier model had 19 degrees of freedom (6 between pelvis and ground; 3, 1, and 1 at each hip, knee and ankle, respectively; 3 at the lumbar joint between trunk and pelvis).

Each ski was discretized into 18 rigid segments (7 rear segments, 1 center segment and 10 front segments) connected by revolute joints. Mass and inertia properties of the ski segments were derived form measurement data of a competitive giant slalom ski. The length of the ski was 2.02 m with a sidecut radius of 32 m and a mass of 2.1 kg. The center segment was firmly affixed to the foot-ski boot segment of the skier model. Rotational spring-damper elements were attached to the revolute joints to incorporate stiffness and damping properties of the skis (Heinrich et al., 2014). Stiffness and damping parameters were derived from laboratory measurements of bending deflection and bending vibration (Mössner et al., 2014). Torsional twist of the skis was neglected because it was shown to be low during turning maneuvers (Yoneyama et al., 2008).

The motion of the skier was actuated by 94 muscles (43 per leg and eight actuating the lumbar joint). The muscle model was based on the OpenSim model of Catelli et al. (2019), since deep squatting and high hip flexion are often encountered during turning maneuvers. In the OpenSim model of Catelli et al. (2019), 80 muscles were includes (40 per leg) actuating the joints of both legs only. To actuate the lumbar joint additionally, we added eight muscles (2 × erector spinae, rectus abdominus, external obliques, internal obliques) to the skier model (Harris et al., 2017). Furthermore, we added three hip muscles (gemelli, pectineus, quadratus femoris) to each leg to increase hip muscle strength based on the musculoskeletal model presented in Harris et al. (2017) focusing on hip musculature.

Muscle activation dynamics was assumed as a first-order process (He, 1991) and models the change in muscle activation as a function of the current active state and the neural excitation of the muscle. The corresponding time constants for muscle activation and deactivation were set to 10 and 40 ms, respectively (Zajac, 1989; Nitschke et al., 2020). Muscle contraction dynamics was modeled in analogy to Nitschke et al. (2020) assuming a three-element Hill-type muscle model, which incorporates the force-length-velocity characteristics of the muscle. Contraction dynamics was formulated in implicit form, which was shown to result in better convergence and increased computational speed (van den Bogert et al., 2011; De Groote et al., 2016).

The dynamics of the whole musculoskeletal skier model was given by the multibody dynamics of the skier and skis, the muscle activation dynamics and the muscle contraction dynamics. The dynamics was formulated in implicit form (van den Bogert et al., 2011) as

$$f\left(x,\dot{x},u\right)=0$$ (1)

where *x* denotes the states of the musculoskeletal skier model, $\dot{x}$ the time derivative of the states and *u* the controls of the musculoskeletal skier model. Specifically, the states *x* of the musculoskeletal skier model were represented by the degrees of freedom and their derivatives of the multibody model of the skier and skis, the projected length of the muscle fibers (=length of the contractile element in the Hill muscle model) and the muscle activations; the controls *u* of the musculoskeletal skier model were represented by the neural muscle excitations of the muscles (van den Bogert et al., 2011).

### 2.2 Ski-Snow Contact Model

We modeled the ski-snow contact using three types of forces acting on each segment of both skis (Mössner et al., 2014). First, we applied a penetration force *F*_*p*_ acting normal to the snow surface. The penetration force was based on an elastic force penetration relation and depended on the penetration depth and speed of the edge of the ski segment orthogonal to the snow surface as well as the edging angle of the ski. The edging angle was defined as the angle between the base surface of the ski segment and the snow surface. Second, we applied a shear force *F*_*s*_ acting orthogonal to the ski edge and parallel to the snow surface. The shear force provided resistance against lateral shearing and depended on the penetration depth of the ski edge. Finally, we applied a frictional force *F*_*f*_ acting antiparallel to the segment’s velocity with a constant friction coefficient *μ* = 0.1 (Heinrich et al., 2014; Mössner et al., 2014).

### 2.3 Experimental Data

To analyze a turning maneuver with the present musculoskeletal model we took measurement data collected by our working group in a previous study (Filippi Oberegger, 2011) where a professional skiing instructor performed a turn to the right with the same giant slalom skies implemented in the musculoskeletal skier model. The movement of the skier was captured by a multi-camera system consisting of three cameras and a frequency of 50 Hz. In a post-processing step 23 landmarks of the skier were manually digitized and the three dimensional coordinates were reconstructed using the direct linear transformation (DLT) algorithm. Given the 23 landmarks we used OpenSim to scale the skier model and used the inverse kinematics tool to compute the kinematics of the skier (i.e., joint angles of skier at the inside and outside leg, joint angles at the lumbar joint and the translation and orientation of the pelvis segment of the skier).

### 2.4 Optimization Framework

Given the musculoskeletal skier model, we used a forward dynamics optimization framework to simulate the movement of the skier, track the experimental data of the skier during the turning maneuver and to compute the joint moments of the skier. Specifically, we formulated a corresponding optimal control problem (i.e., tracking problem). The task of the optimal control problem was to find the states *x* and controls *u* of the musculoskeletal skier model such that a given objective function *J* is minimized (van den Bogert et al., 2011). Specifically, we used the following objective function

$$J=\frac{1}{T}\int_{0}^{T}\underset{\mathrm{tracking}\;\mathrm{error}}{\underset{︸}{w_{1}\|err_{q}{\|}_{2}^{2}}}+\underset{\mathrm{muscle}\;\mathrm{effort}}{\underset{︸}{w_{2}\left(\sum_{i=1}^{\mathrm{nmus}}a_{i}^{2}\right)}}+\underset{\mathrm{regularization}}{\underset{︸}{w_{3}\left(\|\dot{x}{\|}_{2}^{2}+\|\dot{u}{\|}_{2}^{2}\right)}}$$

including a tracking error term, a muscle effort term and a regularization term. Simulation time is denoted by *T*, ‖ − ‖_2_ denotes the Euclidean norm, *nmus* the number of muscles and *w*_1_, *w*_2_ and *w*_3_ are weighting factors.

The first term in the objective function corresponded to the tracking error where *err*_*q*_ denotes the deviation of the degrees of freedom of the skier model (i.e., pelvic translation and rotation, joint angles at the lumbar, hip, knee and ankle joints) and the corresponding measurement data. The second term in the objective function corresponded to muscle effort and was used to resolve muscle redundancy (having more muscles than degrees of freedom). In the literature several criteria have been suggested (Erdemir et al., 2007). One common criterion is to use muscle activation *a* squared as a surrogate for muscle effort. This criterion has been used in a number of studies involving dynamic movement tasks such as jump landing (Laughlin et al., 2011), squatting (Catelli et al., 2019) or cutting (Weinhandl and O’Connor, 2017). Finally, a small regularization term was added with a small weight factor *w*_3_ to enhance convergence by minimizing the derivatives of the states *x* and controls *u* (Nitschke et al., 2020).

The optimal control problem was subjected to constraints due to the dynamics of the musculoskeletal skier model (i.e., muscle activation and contraction dynamics and multibody dynamics of the skier model) as well as lower and upper bounds on the states *x* and controls *u* (van den Bogert et al., 2011; Nitschke et al., 2020).

### 2.5 Model Implementation and Numerical Solution

Solving an optimal control problem is computationally challenging. Recently, however, several efficient computational frameworks have been developed for solving dynamic optimization problems (e.g., Falisse et al., 2019; Nitschke et al., 2020). Similar to the approach of Nitschke et al. (2020) we implemented and solved the optimal control problem in an efficient way. We used MotionGenesis (Motion Genesis LLC, Menlo Park, CA, United States) to generate the equations of motion for the multibody dynamics of the skier model. The equations of motion were exported as C-code and imported in MATLAB *via* the MEX interface. In MATLAB we fused the equations for the multibody dynamics and the muscle contraction and activation dynamics and formulated the optimal control problem (i.e., tracking problem). To solve the optimal control problem, we transformed it into a constrained nonlinear programming problem (NLP) using direct collocation and the implicit Euler formula (van den Bogert et al., 2011). To increase computational speed, we provided analytical derivatives of the objective function and constraints to the NLP solver IPOPT (Nitschke et al., 2020).

### 2.6 Data Analysis

To evaluate the simulation of the turning maneuvers and the associated tracking error, we first calculated the root mean squared difference (RMSD) between the joint angles of the skier derived from the measurement data and the corresponding joint angles of the skier in the simulation. Second, we compared the track of the skier where we used the ankle joint centers of the outside and inside leg as reference points (Supej et al., 2020). Finally, we computed the RMSD between the measured and simulated speed of the skier during the turning maneuver. In the data analysis, we focused on the steering phase of the turning maneuvers, where the skier is subjected to the highest loads (Klous et al., 2012, 2014). Similar to the recent study of Supej et al. (2020), we defined the beginning and end of steering phase when the turn radius of the skier was below the side cut radius of the ski (R = 32 m). After the evaluation we computed the joint moments at the hip, knee and ankle joint of the outside and inside leg as well as the lumbar joint of the skier. Joint moments were represented as internal joint moments and hip flexion, adduction and internal rotation, knee extension and ankle dorsiflexion moments were denoted as positive. To analyze the loading of the knee joints in more detail, we further computed the full 6-DOF intersegmental joint moments and forces at the knee of the outside and inside leg (van den Bogert et al., 2013), respectively, solving the Newton-Euler equations consecutively starting at the ski segments to the shank segment. Since intersegmental joint forces are not the total forces at joint and have limited utility on their own (Derrick et al., 2020), we focused on the analysis of the intersegmental knee joint moments. The intersegmental knee joint moments and forces were represented in the local coordinate system of the shank; the x-axis, y-axis and z-axis referred to the anterior-posterior, superior-inferior and medial-lateral direction.

## 3 Results

### 3.1 Kinematics

**FIGURE 2:**
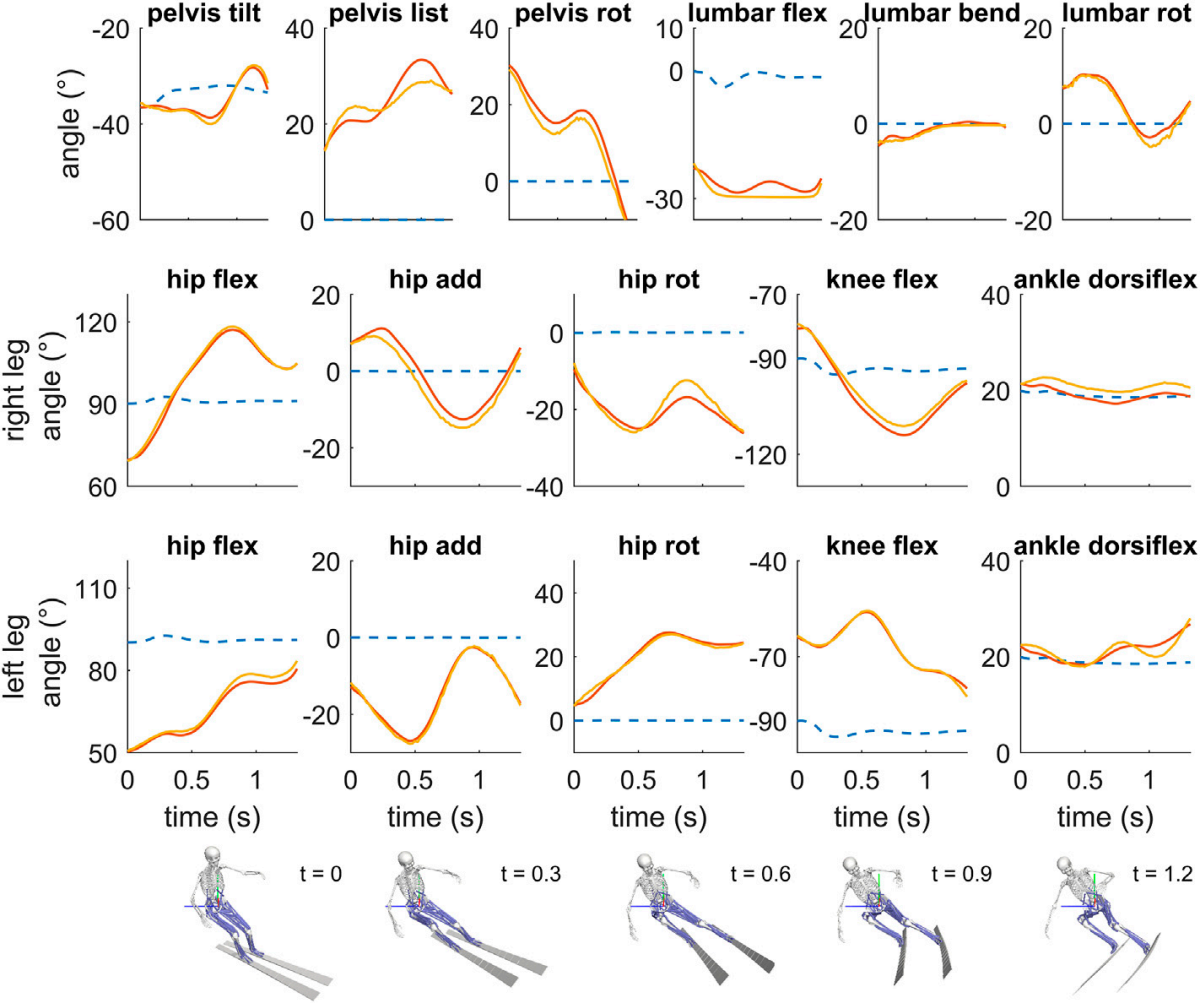
Comparison of the optimized kinematics of the skier (red) during the turning simulation and the corresponding measured kinematic data (yellow). Kinematic data refer to the orientation of the pelvis (tilt, list, rotation) and the joint angles at the lumbar joint (flexion, lateral bending, rotation), hip joint (flexion, adduction, internal rotation), knee joint (flexion) and ankle joint (dorsiflexion). The blue dotted lines represent the kinematics of a straight schussing maneuver, which was used as initial guess in the optimization framework.

In the forward dynamics optimization framework, the turning maneuver could be successfully simulated (an animation of the simulated turning maneuver is provided as Supplementary Material) in about 35 min of computational time on a single core of a workstation (Thinkstation 330, 3.5 GHz E-2146 CPU). In the simulation, the musculoskeletal skier model was able to track the measured kinematic data closely (Figure 2). Specifically, the RMSD between the measured joint angles and the joint angles obtained by the musculoskeletal skier model were in the range from 0.50 to 2.72° (Table 1). The lowest differences were observed for lumbar bending and knee flexion at the outside left leg; the highest differences were observed for pelvis list and hip adduction at the inside right leg.

**TABLE 1:** Root mean squared difference (RMSD) between the measured joint angles of the skier during the turning maneuvers and the corresponding joint angles of the musculoskeletal skier model in the tracking simulation. Minimum and maximum values of the joint angles of the skier during the turning maneuvers are reported, additionally.

	—	RMSD	Minimum	Maximum
Pelvis (deg)			
—	Tilt	0.73	-38.7	-28.3
	List	2.72	14.6	33.4
	Rotation	2.60	-18.4	30.3
Right hip (deg)			
—	Flexion	1.16	69.5	117.1
	Adduction	2.63	-12.6	11.1
	Rotation	2.30	-26.2	-9.5
Right knee (deg)			
—	Flexion	2.03	-114.0	-80.5
Right ankle (deg)			
—	dorsiflexion	2.04	17.2	21.4
Left hip (deg)			
—	Flexion	1.85	50.3	80.6
	Adduction	0.67	-27.0	-2.6
	Rotation	0.92	4.7	27.5
Left knee (deg)			
—	Flexion	0.62	-79.9	-56.1
Left ankle (deg)			
—	Dorsiflexion	1.21	18.3	26.7
Lumbar (deg)			
—	Flexion	2.16	-28.7	-22.8
	Bending	0.50	-4.6	0.4
	Rotation	1.06	-2.8	10.3

**FIGURE 3:**
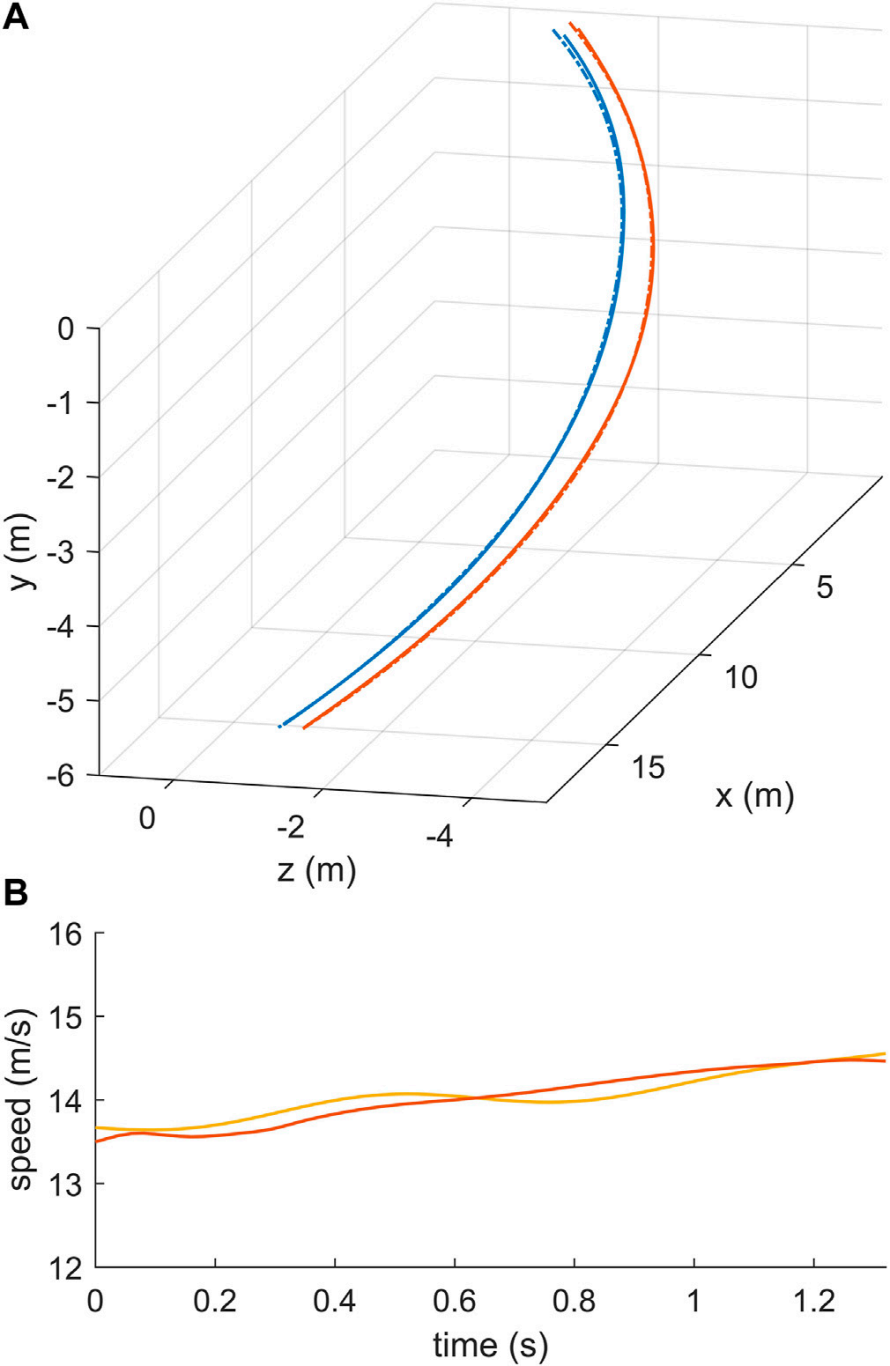
Comparison of the optimized track of the skier in the turning simulation (solid lines) and the corresponding measurement data (dashed lines) in **(A) (B)** shows the measured (solid red) and optimized speed (solid yellow) of the skier.

**FIGURE 4:**
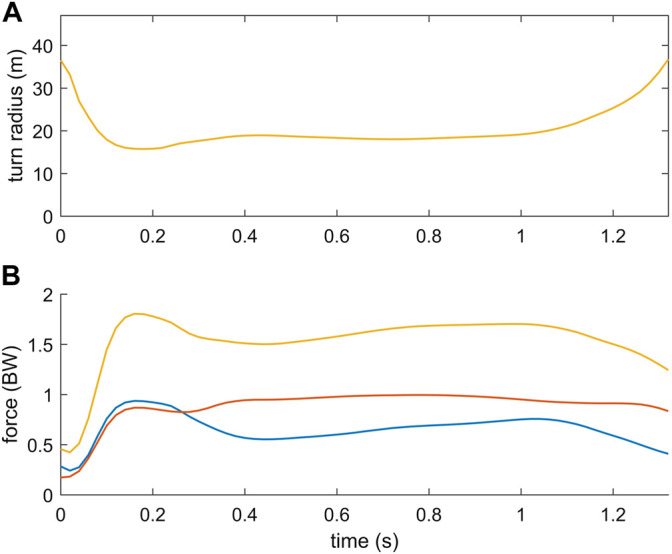
Turn radius of the center of mass of the skier in the turning simulation **(A)** as well as the total ground reaction force, the ground reaction force acting on the outside ski (red) and inside ski (blue) **(B)**.

In addition, the simulated track of the skier and the speed of the skier were in good agreement with the measurement data At the inside and outside leg, the mean deviation between the measured and simulated track was 0.025 and 0.018 m, respectively (Figure 3A). The speed of the skier increased from about 13.5 m/s to 14.5 m/s during the simulated turning maneuver and matched the measured speed with a root mean squared difference of 0.12 m/s (Figure 3B). For the speed comparison, the midpoint between the right and left hip joint center was chosen as the reference point. The turn radius of the center of mass of the skier dropped at the beginning of the steering phase to a minimum of about 16 m and remained almost constant afterwards in the range from 18 to 19 m (Figure 4A).

### 3.2 Ground Reaction Forces

In the simulation of the turning maneuver, the ground reaction forces were higher on the outside leg compared to the inside leg (Figure 4B). Computing the force distribution between the inside and outside leg, about 60% of the total ground reaction force was acting on average on the outside leg. Consequently, the load on the outside leg was on average 50% higher. Peak forces reached 1.00 BW and 0.94 BW on the outside and inside leg, respectively. The local maximum at the beginning of the steering phase was induced by the skier performing an unloading-loading motion after the phase of edge change and the beginning of the steering phase (see animation provided online as Supplementary Material).

### 3.3 Joint Moments

**FIGURE 5:**
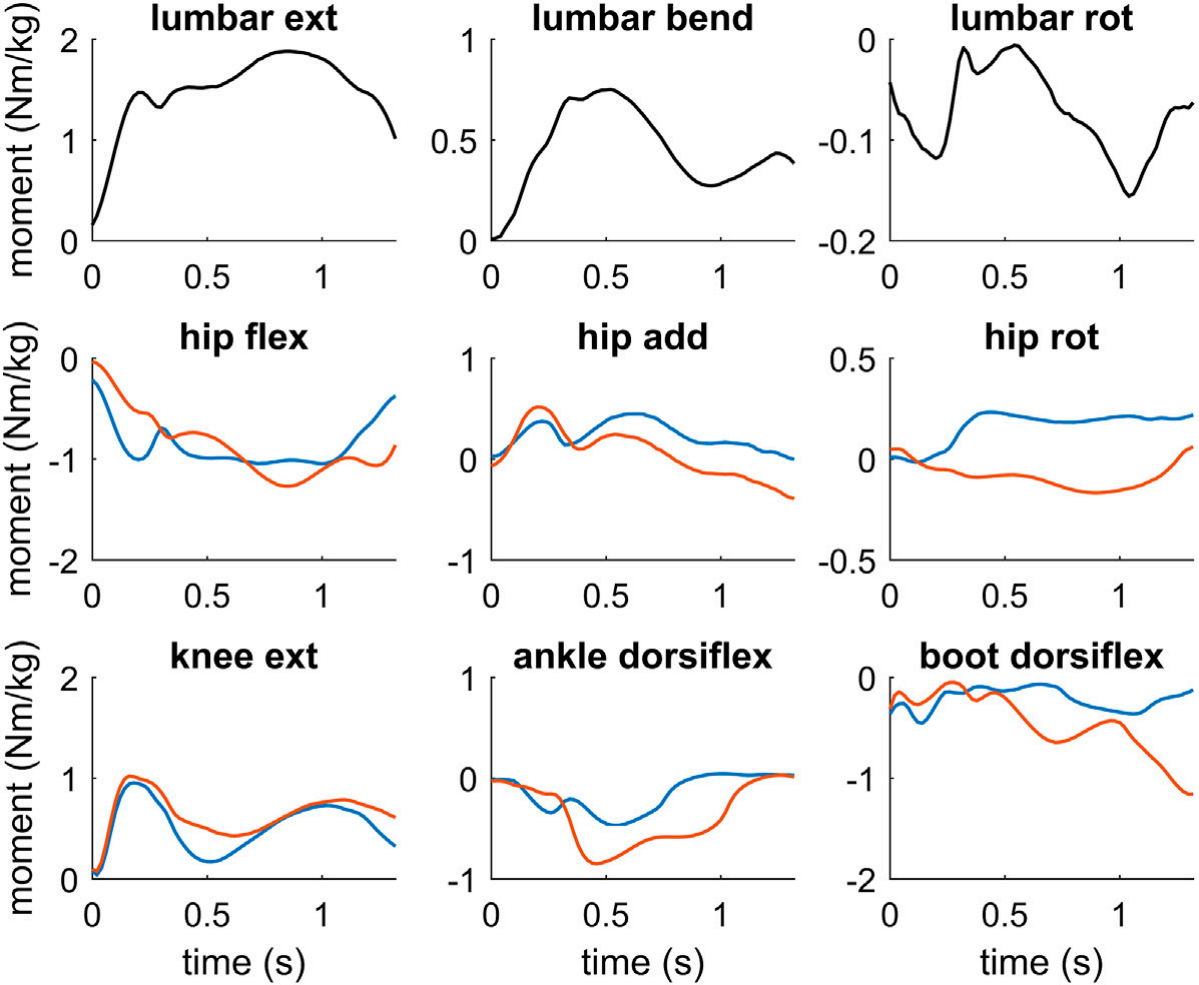
Joint moments at the lumbar joint, hip, knee and ankle joint of the inside leg (blue) and the outside leg (red) as well as the passive joint moment induced by the ski boot at the ankle joint. Joint moments are represented as internal joint moments and hip flexion (hip flex), adduction (hip add) and internal rotation (hip rot), knee extension (knee ext) and ankle dorsiflexion (ankle dorsiflex) moments are denoted as positive. At the lumbar joint, lumbar extension (lumbar ext), lateral bending (lumbar bend) and left rotation (lumbar rot) moments are denoted as positive.

The highest internal joint moments were observed at the lumbar joint with a maximum value of 1.88 Nm/kg for lumbar extension. This was about 2.5 times larger compared to the maximum lumbar bending moment rising to 0.75 Nm/kg and about 12 times larger compared to maximum lumbar rotation moment (Table 2; Figure 5). At the outside leg, the highest internal joint moments corresponded to the hip extension moment with 1.27 Nm/kg, the knee extension moment with 1.02 Nm/kg and the ankle plantarflexion moment with 0.85 Nm/kg. At the inside leg, peak knee and hip extension moments were of similar order compared to the inside leg. The ankle plantarflexion moment and the passive boot moment, however, were 45 and 60% lower, respectively (Table 2; Figure 5).

**TABLE 2:** Peak joint moments at the lumbar joint as well as the hip, knee and ankle joint of the inside right knee and outside left knee, respectively.

Joint moments	—	Minium	Maximum
Right hip (Nm/kg)			
—	Flexion	-1.05	-0.21
	Adduction	0.00	0.45
	Rotation	-0.02	0.23
Right knee (Nm/kg)			
—	Extension	0.04	0.96
Right ankle (Nm/kg)			
—	Dorsiflexion	-0.47	0.04
	ski boot	-0.46	-0.07
Left hip (Nm/kg)			
—	Flexion	-1.27	-0.03
	Adduction	-0.39	0.52
	Rotation	-0.17	0.06
Left knee (Nm/kg)			
—	Extension	0.07	1.02
Left ankle (Nm/kg)			
—	Dorsiflexion	-0.85	0.03
	ski boot	-1.16	-0.05
Lumbar (Nm/kg)			
—	Extension	0.16	1.88
	Bending	0.01	0.75
	Rotation	-0.16	-0.01

Joint moments are represented as internal joint moments and hip flexion, adduction and internal rotation, knee extension, ankle dorsiflexion and lumbar extension, lateral bending and left rotation moments are denoted as positive.

**FIGURE 6:**
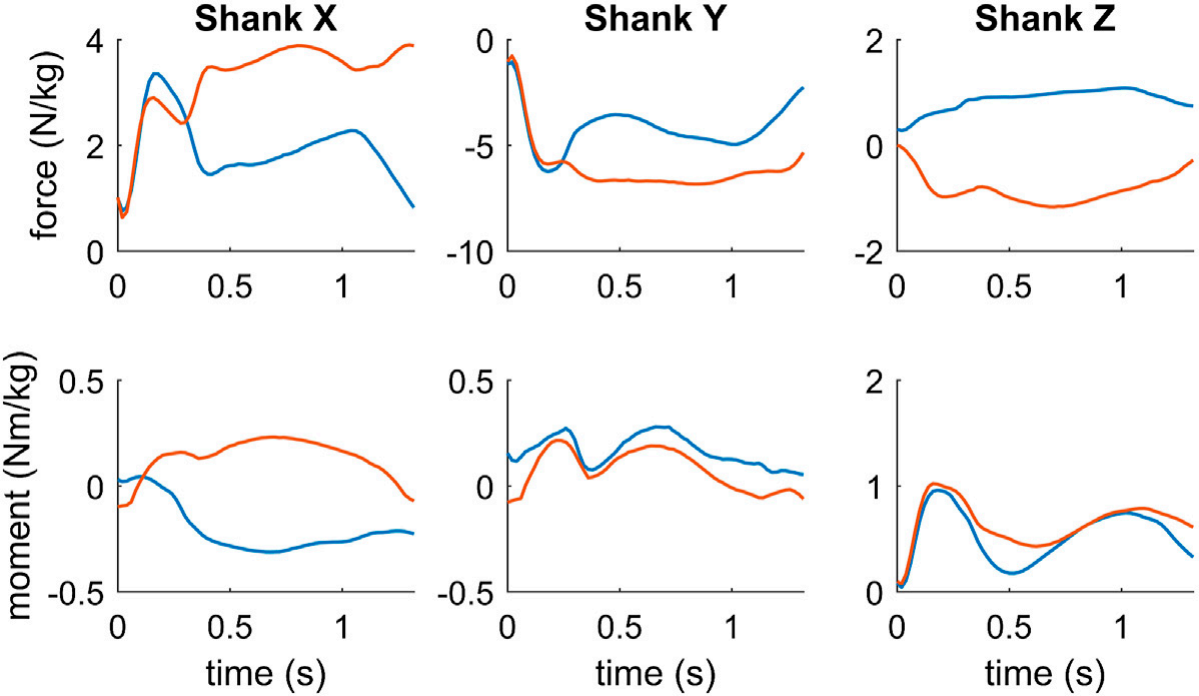
Intersegmental forces and moments at knee joint of the inside leg (blue) and outside leg (red), respectively. Forces and moments are represented in the shank coordinate system, where the *z*-axis, *x*-axis and *y*-axis refer to the medial-lateral, anterior-posterior and superior-inferior direction. Positive joint moments denote an internal knee extension, adduction and internal rotation moment, respectively.

The intersegmental knee joint moments in the frontal plane showed that primarily an internal adduction moment was acting on the knee joint of the outside leg during the turning maneuver (Figure 5), which was mainly induced by the ground reaction force passing laterally to the knee. Correspondingly, the intersegmental mediolateral force at the knee joint pointed medially to counteract the lateral component of the ground reaction force. Contrary, primarily an internal abduction moment acted on the knee joint of the inside leg during the turning maneuver (Figure 6) caused by the ground reaction force passing medially to the knee. Correspondingly, the intersegmental mediolateral force at the knee joint pointed laterally to counteract the medial component of the ground reaction force. Peak internal knee adduction and abduction moments reached 0.23 and 0.31 Nm/kg at the outside and inside leg, respectively (Table 3). In the transverse plane, the rotation moments at the knee ranged between -0.08 and 0.22 Nm/kg at the outside leg with alternating phases of internal and external rotation (Figure 6). At the inside leg, mainly an internal rotation moment was present throughout the turning maneuver with values ranging between 0.05 and 0.28 Nm/kg (Table 3).

**TABLE 3:** Peak intersegmental forces and moments during the turning maneuver at the inside right knee and outside left knee, respectively, represented in the corresponding shank coordinate system.

	—	Minimum	Maximum
Right knee forces (N/kg)			
—	Fx	0.76	3.35
	Fy	-6.23	-1.07
	Fz	0.28	1.08
Right knee moments (Nm/kg)			
—	Mx	-0.31	0.04
	My	0.05	0.28
	Mz	0.04	0.96
Left knee forces (N/kg)			
—	Fx	0.63	3.90
	Fy	-6.83	-0.77
	Fz	-1.16	0.00
Left knee moments (Nm/kg)			
—	Mx	-0.10	0.23
	My	-0.08	0.22
	Mz	0.07	1.02

The *z*-axis, *x*-axis and *y*-axis refer to the medial-lateral, anterior-posterior and superior-inferior direction. Positive joint moments denote an internal knee extension, adduction and internal rotation moment, respectively.

## 4 Discussion

The main objectives of the present study were to 1) develop a three dimensional musculoskeletal simulation model of an alpine skier and 2) apply the musculoskeletal skier model in combination with a forward dynamics optimization framework to estimate dynamically consistent kinematics, ground reaction forces and joint moments during a turning maneuver. The estimation of the joint moments was constrained such that computed joint torques stayed within physiological limits imposed by the musculoskeletal model.

### 4.1 Musculoskeletal Simulation Model

We developed a novel three dimensional musculoskeletal model of an alpine skier with two skis and 53 kinematic degrees of freedom and applied it successfully to simulate and analyze a turning maneuver. To the authors’ knowledge, the present study incorporates the first three dimensional musculoskeletal model for analyzing turning maneuvers in alpine skiing. In previous musculoskeletal simulation studies in alpine skiing only two dimensional models were developed (Gerritsen et al., 1996; Heinrich et al., 2014, 2018). These models were applied to analyze jump landing maneuvers in downhill skiing in the sagittal plane and possible risk factor for an injury of the anterior cruciate ligament. Developing a three dimensional musculoskeletal simulation model is computational challenging due to the increased model complexity and consequently the high computational cost involved for the optimization. To reduce the computational cost, we employed recent advances in musculoskeletal modeling and simulation. In particular, we formulated the system dynamics in implicit form (van den Bogert et al., 2011) and used direct collocation with analytical derivatives of the system dynamics to solve the underlying dynamic optimization problem (Nitschke et al., 2020).

The simulation of the turning maneuver was based on a forward dynamics optimization framework (i.e., forward assisted data tracking), where measured kinematic data of an alpine skier performing a turning maneuver were tracked by the musculoskeletal skier model. In the simulation of the turning maneuver, the experimental data of the skier could be tracked closely with a RMSD below 3° at all joints. This RMSD is considered to be low, because it is well within the precision of current mobile measurement devices such as inertial measurement units (IMU) based systems (Fasel et al., 2018b) or machine learning techniques (Ostrek et al., 2019). Additionally, the track and the speed of the skier could be tracked well. Comparing the inside and outside leg, higher errors were detected at the inside leg. The higher errors could have been caused by the higher hip and knee flexion on the inside leg during the turning. This might have complicated the manual digitization process due to occlusions and consequently reduced the accuracy of the corresponding measurement data.

In the forward dynamics optimization framework, kinematic data obtained by video-based stereophotogrammetry and a multi-camera setup were used as input data. IMU based systems in combination with a Global Navigation Satellite System (GNSS) or computer vision and human pose estimation have been shown to be promising approaches for capturing the kinematics of a skier during turning maneuvers on the ski slope (e.g., Fasel et al., 2018b; Ostrek et al., 2019). Since only measured kinematic data are mandatory in the forward dynamics optimization framework (see also next section), the present musculoskeletal simulation model might also be combined with either of these approaches in future research without the need to provide ground reaction force data captured by mobile force platforms or pressure insoles. This might open up new opportunities regarding the analysis of the loading of the skier during turning maneuvers in the natural environment if only kinematic data are available. If measured ground reaction force, however, are additionally available as input data, these data might also be tracked in the optimization framework. Specifically, in the objective function of the optimization framework the deviation of the simulated and measured ground reaction forces might be taken into account to track the measured ground reaction forces as good as possible (van den Bogert et al., 2011; Nitschke et al., 2020).

### 4.2 Forward Dynamics Optimization Framework

In the present study we used a forward dynamics optimization framework to compute the joint moments during a tuning maneuver. In the literature there are only a few studies analyzing the loading of the skier during turning maneuvers dynamically (Klous et al., 2012, 2014; Hirose et al., 2013; Lee et al., 2017). However, in all of these studies the authors used an inverse dynamics approach to compute the joint moments at the lower extremities. Compared to the classical inverse dynamics analysis, the present framework has four major advantages.

First, using a forward dynamics optimization framework the obtained kinematics of the skier as well as the corresponding ground reaction forces are dynamically consistent. Consequently, no residual forces and torque have to be added such that the equations of motion of the skier are satisfied (Bailly et al., 2021). This is important since residual forces and moments do not exist in reality and affect the computation of kinetic variables such as joint moments, power and work (Hatze, 2002; Faber et al., 2018). Contrary, using an inverse dynamics approach the validity of the computed joint moments is impaired depending on the size of the introduced residual forces and moments (Faber et al., 2018).

Second, optimization of a forward dynamic model with data tracking and effort minimization also has the advantage that the data being tracked can be any number of variables. The number of measurements can be overdetermined (more measurements than kinematic degrees of freedom and external loads), or underdetermined (fewer measurements than kinematic degrees of freedom and external loads). This makes it possible to perform a full dynamic analysis without external loads (i.e., ground reaction forces) provided for example by instrumented skis, as demonstrated previously in a planar analysis of jump landing in skiing (van den Bogert et al., 2011) or in the present three dimensional analysis of a turning maneuver in skiing. In contrast, classical inverse dynamic analysis requires the measurement of all kinematic degrees of freedom and external loads, not more and not less.

Third, the computed joint moments are less sensitive to errors in the measurement data, since only the measured kinematic data (i.e., joint angles, pelvic translation and orientation) are tracked in the simulation. First or second derivatives of the measured kinematic data are not required. Contrary, inverse dynamics requires the second derivative of the measured kinematic data, which amplifies measurement errors (Cahouët et al., 2002; Pàmies-Vilà et al., 2012).

Fourth, the computed joint moments are constrained to stay within physiological limits. The physiological limits were induced by the musculoskeletal model, which included a three element Hill-type muscle model with activation and contraction dynamics. Contraction dynamics incorporates the force-length-velocity characteristics of the muscle, the active state as well as the maximum isometric force (van den Bogert et al., 2011) and limits the acting muscle forces and the corresponding joint moments. In addition, the activation dynamics limits the change of the corresponding joint moments, incorporating time constants for muscle activation and deactivation in the dynamics equation (Bailly et al., 2021).

### 4.3 Joint Moments

Based on the musculoskeletal model and the forward dynamics optimization framework we computed the joint moments at the lumbar joint as well as the hip, knee and ankle joints of the outside and inside leg of the skier during the turning maneuver. At the outside leg, highest lower-limb joint moments were identified at the hip joint (1.27 Nm/kg, hip extension), followed by the knee joint (1.02 Nm/kg, knee extension) and ankle joint (0.85 Nm/kg, ankle plantarflexion). Knee and hip extension moments were similar at the outside and inside leg, although the ground reaction forces were on average about 50% higher on the outside leg. This can be explained by the increased knee and hip flexion on the inside leg, which required higher activation of the knee and hip extensor muscles. The ankle plantarflexion moment and also the passive boot moment were lower on the inside leg, which indicated that the skier was pushing more against the shaft of the ski boot at the outside leg.

In accordance with the present study, the hip extension moment was reported as the highest joint moment in the kinetic studies of Hirose et al. (2013) and Lee et al. (2017) analyzing turning maneuvers in skiing based. In both studies, the authors used on an inverse dynamics approach to compute the joint moments given kinematic data from an IMU based system and measured ground reaction forces. Specifically, in the study of Lee et al. (2017), the peak knee extension moment (0.5 Nm/kg) and ankle plantarflexion moment (1.1 Nm/kg) were roughly of the same order of magnitude as in the present study. Strikingly, however, the peak hip extension moment reached 6 Nm/kg. In the study of Hirose et al. (2013), the hip extension moment reached 12 Nm/kg assuming a mass of the skier of 75 kg. Compared to the present study, these reported peak hip extension moments are a factor of 5 and 10, respectively, higher and are likely to be unrealistic high. In particular, these values exceed reported maximum voluntary joint moments by about 70 and 240%, respectively, if we take the data of the age group 19–25 in the study of Anderson et al. (2007) as reference data and add one standard deviation to the mean value. Unfortunately, Hirose et al. (2013) and Lee et al. (2017) did not present any information about the external forces acting on the skier (i.e., ground reaction forces or speed and turn radius of the skier) or the kinematics of the skier, which makes a more detailed comparison impossible. On the other hand, similar hip extension moments as in the present study were reported in the study of van den Bogert et al. (1999). Nine male subjects were instrumented with a 12-channel accelerometer system mounted on the upper body and the hip extension moment was computed through an inverse dynamics analysis. The authors reported an average peak hip extension moment of 1.75 Nm/kg during long turns on a flat slope assuming a mean mass of the skiers of 75 kg. The skiers were asked to load the outer ski only and lift the inner ski, which shifted the entire load to the outer ski. In contrast, only 60% of the external load was acting on the outside leg of the skier during the turning maneuver in the present study.

The internal knee joint moments showed that in the frontal plane primarily an adduction moment was acting on the knee joint of the outside leg in combination with alternating phases of an internal and external rotation moment in the transverse plane as well as a knee extension moment. Klous et al. (2012) studied the loading of the knee joint during a carved and skidded turn. The turn radius of the skier was about 10 m in both turns, while the mean speed during the carving turn was higher than during the skidded turn (13.9 m/s vs 10.4 m/s). The authors determined peak knee extension moments up to 8.35 and 4.07 Nm/kg for the skidded and carved turn, respectively, based on an inverse dynamics approach. In addition, they computed peak adduction moments up to 5.70 and 5.75 Nm/kg in the frontal plane and external rotation moments up to 6.85 and 2.75 Nm/kg in the transverse plane for the skidded and carved turn. All of these values are significantly higher compared to the values of the present study. The differences might be explained in part by the decreased turn radius of the skier in the study of Klous et al. (2012), which induced a higher external loading onto the skier. However, due to measurement noise and the limitations of the inverse dynamics approach, the computed joint moments might have been overestimated exceeding physiologically plausible values. For example, if we compare the peak knee extension moments with literature data such as maximum voluntary knee extension moments (Anderson et al., 2007) or maximum isometric knee extension moments (Pincivero et al., 2004; Domire et al., 2011), these values are unrealistic high. In addition, the reported knee abduction moments are about three times higher than the assumed injury threshold of 125 Nm valgus moment (= 1.67 Nm/kg assuming a mass of 75 kg) in the study of McLean et al. (2008), although they did not investigate an injury prone situation.

Interestingly, in the present study the peak joint moment at the lumbar joint exceeded the peak values at the lower limbs during the turning maneuver. In particular, the lumbar extension moment rose up to 1.88 Nm/kg, while the lateral bending and rotation moments were remarkably lower. Consistent with the results of the present study, the highest joint moment was observed at the lumbar joint for lumbar extension in the study of Hirose et al. (2013). High values at the lumbar joint imply that the lower back of the skier is subjected to high loads during turning maneuvers. These high values may be linked to lower back pain, which is a common overuse injury in alpine skiing (Spörri et al., 2018). Furthermore, it has been reported that the combination of lumbar flexion, lateral bending and axial rotation amplifies the loading at the lower back (Spörri et al., 2018). Further studies, however, and a more sophisticated model of the lower back are necessary to quantify the internal loading at the lower back in more detail.

### 4.4 Limitations

Some limitations of the present study have to be mentioned. First, in the simulation of the turning maneuver we did not track the movement of the arms of the skier. The reason was to reduce the complexity of the model and to decrease computational time, which is one of the big challenges in three dimensional musculoskeletal simulations. However, since we included the mass and inertia properties of the arm in the model and assumed a mean posture of the arms in front of the skier, the impact on the computed joint moments is expected to be low.

Second, we did not implement a detailed spine model, but used a single lumbar joint at the lower back. Consequently, the present simulation model is expected to provide only basic features regarding the loading of the lower back of the skier turning maneuvers. While these basic features might contribute to the understanding of lower back pain, which is a common overuse injury in alpine skiing (Spörri et al., 2018), a more detailed spine model might provide further insight.

Third, in the present simulation study we analyzed data of a professional skiing instructor performing a giant slalom turning maneuver. Changing the characteristics of equipment, the present simulation model might also be used to analyze turning maneuvers in other disciplines such as slalom, super-G (super giant slalom), or downhill skiing. Furthermore, the present simulation model might also be used to analyze jump landing maneuvers in super-G and downhill, which have been identified as a common situation leading to injury (Gilgien et al., 2014). Regarding jump landing maneuvers the present simulation model might extend the current knowledge derived from two dimensional simulation models (Heinrich et al., 2014) regarding the loading of the skier in the frontal and transverse plane.

## 5 Conclusion and Outlook

In the present study we developed a novel three dimensional musculoskeletal simulation model to analyze the kinematics and kinetics (i.e., the intersegmental moments at the knee joint) of a skier during turning maneuvers. While the focus of the present study was on the joint moments acting on the skier, the present musculoskeletal model might also be applied to analyze muscle forces and further characteristics related to muscle function such as muscle length change, muscle contraction velocity, muscle power and muscle work (van den Bogert et al., 2013). Kinematic data captured by a multi-camera setup were used as input data. In future applications, the present simulation model might also be used in combination with kinematic data obtained by mobile measurement devices (i.e., IMU and GNSS based systems) or machine learning techniques (i.e., human pose estimation) providing additional insight into the loading of the skier during turning maneuvers.

## Data Availability

The original contributions presented in the study are included in the article/Supplementary Material, further inquiries can be directed to the corresponding author.
